# SIV Infection of Lung Macrophages

**DOI:** 10.1371/journal.pone.0125500

**Published:** 2015-05-01

**Authors:** Yue Li, Guobin Kang, Lijie Duan, Wuxun Lu, Michael G. Katze, Mark G. Lewis, Ashley T. Haase, Qingsheng Li

**Affiliations:** 1 Nebraska Center for Virology, School of Biological Sciences, University of Nebraska-Lincoln, Lincoln, Nebraska, United States of America; 2 College of Life Sciences, Nankai University, Tianjin, People’s Republic of China; 3 Department of Microbiology, University of Minnesota Medical School, Minneapolis, Minnesota, United States of America; 4 Washington National Primate Research Center, Seattle, Washington, United States of America; 5 Department of Microbiology, School of Medicine, University of Washington, Seattle, Washington, United States of America; 6 BIOQUAL, Inc., 9600 Medical Center Drive, Rockville, Maryland, United States of America; University of Pittsburgh Center for Vaccine Research, UNITED STATES

## Abstract

HIV-1 depletes CD4+ T cells in the blood, lymphatic tissues, gut and lungs. Here we investigated the relationship between depletion and infection of CD4+ T cells in the lung parenchyma. The lungs of 38 Indian rhesus macaques in early to later stages of SIVmac251 infection were examined, and the numbers of CD4+ T cells and macrophages plus the frequency of SIV RNA+ cells were quantified. We showed that SIV infected macrophages in the lung parenchyma, but only in small numbers except in the setting of interstitial inflammation where large numbers of SIV RNA+ macrophages were detected. However, even in this setting, the number of macrophages was not decreased. By contrast, there were few infected CD4+ T cells in lung parenchyma, but CD4+ T cells were nonetheless depleted by unknown mechanisms. The CD4+ T cells in lung parenchyma were depleted even though they were not productively infected, whereas SIV can infect large numbers of macrophages in the setting of interstitial inflammation without depleting them. These observations point to the need for future investigations into mechanisms of CD4+ T cell depletion at this mucosal site, and into mechanisms by which macrophage populations are maintained despite high levels of infection. The large numbers of SIV RNA+ macrophages in lungs in the setting of interstitial inflammation indicates that lung macrophages can be an important source for SIV persistent infection.

## Introduction

Human immunodeficiency virus type 1 (HIV-1) depletes CD4+ T cells in blood, secondary lymphatic tissues, gut and lungs by mechanisms such as the cytopathic effects of infection, immune recognition and killing of infected cells. In the lung, CD4+ T cell count in bronchoalveolar lavage (BAL) quickly declines in acute HIV-1 and SIV infection [1–4], and indeed this measurement is commonly used as a surrogate for mucosal CD4+ T cell depletion in acute HIV-1 infection. However, the relationship between infection in the lung parenchyma and decreased CD4+ T cells in BAL has not been determined. To that end, we explored the role of infection in depletion of CD4+ T cells in the lung parenchyma of SIV-infected macaques from the early to later stages of infection. We found that macrophages, but not CD4+ T cells, are the principal host cells that SIV infects. Relatively few macrophages or CD4+ T cells are infected except in the setting of lung interstitial inflammation where we found large numbers of SIV RNA+ macrophages. Even in this setting, SIV RNA+ CD4+ T cells were mainly localized outside of the parenchyma and in broncho-alveolar lymphatic tissue (BALT). Despite productive infection of alveolar macrophages, macrophage populations were preserved, but CD4+ T cell populations were depleted even though they were not productively infected. Thus, mechanisms other than direct infection are responsible for the depletion of CD4+ T cells in BAL in acute SIV infection [2, 5].

## Materials and Methods

### Ethics Statement

This study was reviewed and approved by the Institutional Animal Care and Use Committee (IACUC) at the University of Nebraska-Lincoln, BIOQUAL Inc., Washington National Primate Research Center, and California National Primate Research Center. The care and husbandry of all rhesus macaque were provided in compliance with The Guide for the Care and Use of Laboratory Animals, the Animal Welfare Act, the PHS Policy on Humane Care and Use of Laboratory Animals, and applicable local, state, and federal laws. The comprehensive veterinary care program includes: disease detection and surveillance, prevention, diagnosis, treatment, and resolution; monitoring and promoting physical and psychological well-being of all animals. Monkeys were housed socially, if possible. Monkeys had access to food, water, light at 12-hour cycle and were provided with enrichment toys. Animals were sedated with ketamine for all technical procedures and were fully anesthetized for SIV inoculation and euthanasia under the directions of the attending veterinarians.

### Virus and Animals Inoculation

The lungs of 44 adult Indian rhesus macaques (*Macaca mulatta*) were studied. The animals came from three studies and were housed at BIOQUAL Inc., Washington National Primate Research Center, and California National Primate Research Center. The animals were maintained in accordance with the Guide for the Care and Use of Laboratory Animals. Six macaques were SIV naïve, 29 were acutely infected, and 9 were chronically infected. All animals were free of simian retrovirus type D, simian T-lymphotrophic virus type 1, and herpes B virus. Macaques were inoculated SIVmac251 at a single dose of 3.4×10^4^ TCID_50_ intrarectally or 5.0×10^5^ TCID_50_ intravaginally twice at four hours interval, respectively, and euthanized at designed time points. The lung tissues were collected, fixed in 4% paraformaldehyde for 4–6 hours and embedded in paraffin.

### Plasma Viral RNA Measurements

Virus load in EDTA-treated plasma was quantified using real-time RT-PCR assay based on amplification of conserved sequences in SIV *gag* gene [6]. The limit of detection for this assay was 30 viral RNA copy equivalents per ml plasma.

### Detection of SIV RNA+ Cells in Lungs Using *In Situ* Hybridization

SIV RNA+ cells in the lungs were detected using *in situ* hybridization as previously described [7]. Briefly, 6-μm sections were cut and adhered to silanized slides. After de-paraffinization in xylene, rehydration in PBS and permeabilization by treating the sections with HCl, digitonin and proteinase K, the sections were acetylated and hybridized to ^35^S-labelled SIV-specific riboprobes. After washing and digestion with RNases, sections were coated with nuclear track emulsion, exposed, developed and counterstained with hematoxylin and eosin staining. SIV RNA+ cells were enumerated in sections of defined areas using Aperio ImageScope software (Aperio ePathology Solutions, CA).

### Detection of CD4+ T Cells and Macrophages in Lungs Using Immunohistochemical Staining

CD4+ T cells and CD68+ macrophages were detected using IHCS [7]. Briefly, 6-μm sections were cut and adhered to silanized slides from 4% paraformaldehyde fixed and paraffin embedded tissues, and stained with mouse anti-human monoclonal antibody to CD4 (1F6, 1:60 dilution, Leica Microsystems) or CD68 (KP1, 1:200 dilution, DAKO North American, Inc., Carpinteria, CA) for macrophage assay. Sections on slides were pretreated in a Presto pressure cooker (National Presto Industries, Eau Claire, WI) in 1 mM EDTA (pH 8.0) for 35 seconds to unmask CD4 antigen or in 98°C water bath in 10 mM citrate buffer (pH 6.0) for 15 minutes to unmask CD68 antigen. The sections were sequentially treated with aqueous hydrogen peroxide, blocked with normal horse serum, and incubated with primary antibody overnight at 4°C. The stained color was developed by using the Vectastain Mouse-IgG Peroxidase ABC kit (Vector Laboratories, Lumigen Inc. Southfield, MI) and diaminobenzidine (DAB) as substrate in DAB Enhancing Solution (Vector Laboratories, Burlingame, CA). The sections were counterstained with hematoxylin.

### Combined Immunohistochemistry Staining and *In Situ* Hybridization

Combined IHCS and ISH were performed as described previously [7]. Briefly, tissue sections on slides were incubated at 98°C for 15 minutes in 10mM citrate buffer (pH 6.0) for antigen retrieval, hybridized, washed and digested with RNases, incubated with antibody against T cell marker (anti-CD3, SP7, 1:100 dilution, Lab Vision/NeoMarkers, CA) or macrophage marker (anti-CD163, NCI-Cd163, 1:100 dilution, Novocastra Reagents-Leica Biosystems, IL), and then stained with DAB with the Dako Envision and Peroxidase kit (Dako North America, Inc., Carpinteria, CA). After washing, the sections were coated with nuclear track emulsion, exposed, developed and counterstained with hematoxylin.

### Quantitation of CD4+ T cells, Macrophages, and SIV RNA+ Cells in Lungs

Immunochemically stained or ISH tissue sections on slide were digitized using Scanscope (Aperio ePathology Solutions, CA) and the cells of CD4+, CD68+ or SIV RNA+ were quantified using Spectrum Plus analysis program (Version 9.1, Aperio ePathology Solutions, CA) as described [8]. Briefly, a scanned digital slide was opened in ImageScope and pulmonary areas were selected with ImageScope drawing tools for analysis. The lung parenchyma and alveolar space in the digitized slide were separated using a positive pixel count algorithm in the Spectrum Plus analysis program by setting a threshold to measure the parenchyma area only. The cells were quantified by using a positive pixel count algorithm in the Spectrum Plus analysis program. The parameters of the algorithm were manually and accurately tuned to match the positive markup image of DAB staining over background. Once the parameters were set, the algorithm was applied automatically to all digital slides to measure the number of CD4+ or CD68+ cells.

### Statistics

Statistical analysis was conducted with a nonparametric Mann-Whitney U test using Graphpad Prism software (Graphpad software, San Diego, CA, USA). P value < 0.05 was considered significant.

## Results

### SIV infected cells in the lungs in early and later stages of infection

**Fig 1:**
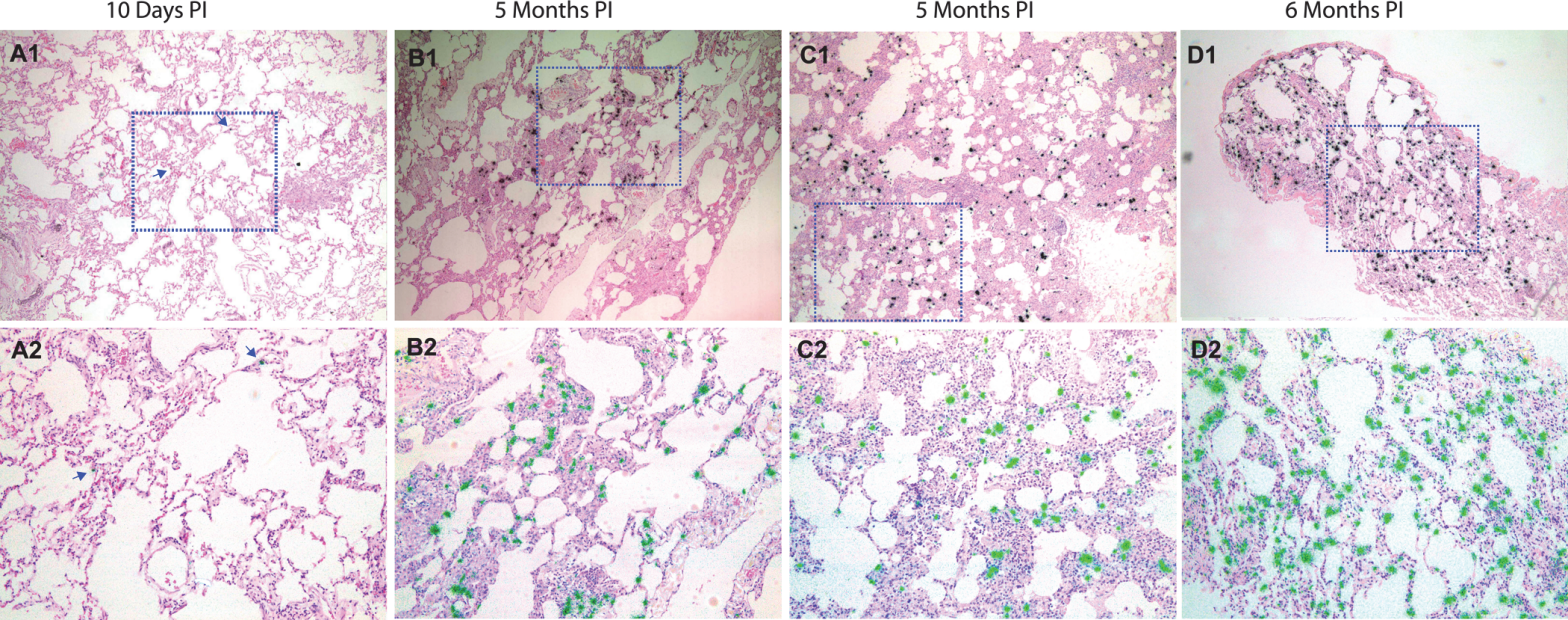
SIV vRNA+ cells increase in the lungs of chronically infected macaques as compared with early infection. SIV vRNA+ cells were detected using *in situ* hybridization, and appeared as black dots in transmitted light and green dots under epipolarized light. There were a few vRNA+ cells in the lung tissues during acute infection (A1, A2) and large numbers of vRNA+ cells in the lung during chronic infection (B1-D2).

We examined the lungs of 6 SIV-uninfected control rhesus macaques and 38 SIV-infected rhesus macaques (Table 1) to characterize SIV productive infection in the lungs from early to later stages of infection. The infected animals were inoculated via vaginal or rectal mucosal routes with high-dose SIVmac251 and sacrificed in the acute (1–14 days PI) (29 animals) or later stages of infection (3–6 months PI) (9 animals). Plasma viral RNA (vRNA) in each animal was determined by real-time RT-PCR and vRNA+ cells in the lung tissues were detected by using *in situ* hybridization (ISH, Table 1). Plasma viral load in these infected monkeys showed similar kinetics as described previously [7], with the viral peak at 10–14 days PI. At 1–4 days PI, no SIV RNA+ cells were detected in the lungs (Table 1) and other distal lymphatic tissues (data not shown), but by 6 days PI, small numbers of vRNA+ cells were detected in lungs of all six macaques, and SIV vRNA+ cells were detected in the lungs of 17 macaques sacrificed at 6–12 days PI (Table 1, Fig 1A1–1A2). The frequencies of vRNA+ cells were much higher in the lungs of 5 out of 9 macaques at 3–6 months PI (Table 1, Fig 1B1–1D2) as compared with all the other macaques examined. Since mycobacterial co-infection can significantly increase HIV-1 and SIV replication [9, 10], we examined but found no evidence of co-infection in these cases using acid fast staining (data not shown). Rather, the SIV RNA+ cells were associated with lung interstitial inflammation, as has been previously reported [11, 12]

**Table 1:** SIV vRNA+ Cells in the Lungs of Macaques Infected with SIV^1^.

Animal ID	DPI^2^ at Necropsy	SIV Inoculation Route	plasma VL at Necropsy^3^	vRNA+ cells in lungs^4^
***Uninfected Control***				
Rh4974	0	No	Negative	-
Rh4975	0	No	Negative	-
Rh4977	0	No	Negative	-
RhM097	0	No	Negative	-
RhM180	0	No	Negative	-
RhM179	0	No	Negative	-
***Acute***				
A1	1	mac251, IR	Negative	-
A2	1	mac251, IR	Negative	-
B1	2	mac251, IR	Negative	-
B2	2	mac251, IR	Negative	-
C1	3	mac251, IR	Negative	-
C2	3	mac251, IR	Negative	-
C3	3	mac251, IR	Negative	-
Rh4971	3	mac251, IR	Negative	-
Rh4972	3	mac251, IR	Negative	-
Rh4973	3	mac251, IR	Negative	-
Rh5043	4	mac251, IR	Negative	-
Rh5044	4	mac251, IR	Negative	-
Rh4809	6	mac251, IR	Positive	+
Rh5052	6	mac251, IR	Positive	+
Rh5053	6	mac251, IR	Positive	+
RhL864	6	mac251, IR	Positive	+
30991	6	mac251, IVAG	Positive	+
31523	6	mac251, IVAG	Positive	+
29608	7	mac251, IVAG	Positive	+
34998	8	mac251, IVAG	Positive	+
28013	9	mac251, IVAG	Positive	+
24818	10	mac251, IVAG	Positive	+
Rh4976	10	mac251, IR	Positive	+
Rh4978	10	mac251, IR	Positive	+
Rh4979	10	mac251, IR	Positive	+
E1	12	mac251, IR	Positive	+
E2	12	mac251, IR	Positive	+
E3	12	mac251, IR	Positive	+
E4	12	mac251, IR	Positive	++
***Chronic***				
F1	12 weeks	mac251, IR	Positive	+
F2	12 weeks	mac251, IR	Positive	++
F3	12 weeks	mac251, IR	Positive	+
F4	12 weeks	mac251, IR	Positive	++
29459	5 months	mac251, IVAG	Positive	++++
23756	5 months	mac251, IVAG	Positive	++++
25301	5 months	mac251, IVAG	Positive	+
27361	6 months	mac251, IVAG	Positive	+
25479	6 months	mac251, IVAG	Positive	++++

^1^Totally 38 infected and 6 uninfected rhesus macaques were used in this study, and vRNA+ cells were quantified by ISH.

^2^DPI = Days post infection.

^3^Virus load in plasma was detected by real-time RT-PCR.

^4^SIV vRNA^+^ cells in lungs were detected using ISH with SIV-specific riboprobes:

−ith SIV-s^+^ cells/mm^2^

+, 5–50 vRNA^+^ cells/mm^2^

++, 51–100 vRNA^+^ cells/mm^2^

++++, >200 vRNA^+^ cells/mm^2^.

### Lung macrophages are primary target cells for SIV

**Fig 2:**
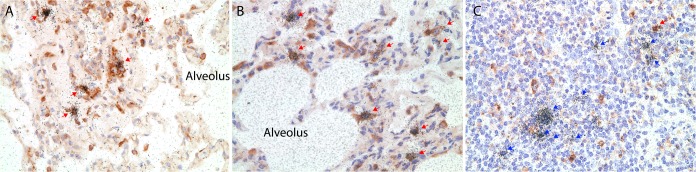
Macrophages are the main SIV RNA+ cells in the lungs from very early (A) at 10 days PI to chronic infection at 5 months PI (B). SIV vRNA+ macrophages were distinguished from T cells by immunohistochemical staining for CD163. The red arrows indicate CD163+ vRNA+ cells identified by the overlying collection of silver grains (black dots); blue arrows indicate SIV RNA+ cells that are not macrophages in lung associated lymphatic tissue (C).

To define the cell types that SIV preferentially infects in lungs, vRNA+ cells were characterized using the combined IHCS and ISH. Macrophages were the main target cells in lung parenchyma in both acute (Fig 2A) and later stages of infections (Fig 2B), but were not the major cell type infected in broncho-associated lymphatic tissues (Fig 2C). The CD4+ T cells in the lung parenchyma were not infected in these cases, but CD4+ T cells were the major cell type infected with SIV in broncho-associated lymphatic tissue examined with the combined ISH and IHCS using CD3 antibody (data not shown).

### CD4+ T cells but not macrophages are depleted in lungs

**Fig 3:**
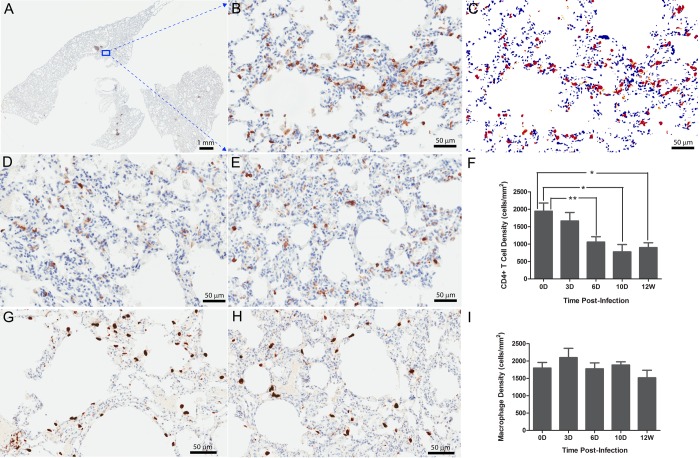
The depletion of CD4+ T cells, but not macrophages, in lung tissues during SIV infection. CD4+ T cells (A-F) and CD68+ macrophages (G-I) in the lung tissues of uninfected and infected macaques detected using immunohistochemical staining and quantified using Aperio Spectrum Plus analysis program. The A-E are representative images of CD4+ T cells in uninfected lung tissues (A-C) and, infected at 10 days (D) and 12 weeks (E) PI respectively. (A) Digitized whole tissue section. (B) Magnified image from the box in the Fig 3A, where the brown corresponds to the stained CD4+ T cells. (C) The red/yellow marked-up regions correspond to the stained CD4+ T cells in the Fig 3B used for quantification with Aperio Spectrum Plus analysis program. The G-H are the representative images of pulmonary CD68+ macrophages in uninfected lung tissues (G) and infected at 12 weeks PI(H). The Fig 3F and 3I are the histograms of CD4+ T cell and macrophage quantification in lung tissues, respectively. X-axis shows the time of infection at 0 (n = 6), 3 (n = 6), 6 (n = 6), 10 (n = 4) days and 12 weeks (n = 4) PI, and y-axis shows the cell number expressed as per square millimeter of lung tissue. *Indicates significant differences from controls (*P<0.05, **P<0.01). Statistical analysis of cell amount per mm^2^ tissue was performed with non-parametric Mann-Whitney U test.

Despite the predominance of infection in macrophages, CD4+ T cells, but not macrophages were depleted in lungs. We quantified CD4+ and CD68+ cells in the lungs of uninfected macaques (as baseline controls) and macaques at various stages of SIV infection (Fig 3) by staining and quantitative image analysis (QIA) (Fig 3A–3C). In uninfected animals, there were 1941±371 CD4+ T cells/mm^2^ (Fig 3B), but as early as 6 days PI, lung CD4+ T cells had decreased significantly (1056±364 cells/mm^2^, p<0.05, non-parametric Mann-Whitney U test) and reached a nadir at 10 days PI (777±400 cells/mm^2^, p<0.05, Fig 3D) and remained depleted through 12 weeks post infection (899±280 cells/mm^2^, p<0.01, Fig 3E and 3F). In contrast, we found no significant change in the number of alveolar and interstitial CD68+ macrophages in acutely (p>0.05, day 3, 6 or 10 PI groups as compared with the control group, respectively,) and chronically (p>0.05, 12 weeks PI group versus control group) infected macaques (Fig 3G–3I).

## Discussion

In this study, we systematically examined the infected cell types and the depletion of CD4+ T cells in the lungs of SIV infected rhesus macaques in the very early to later stages of infection. We found that macrophages were the major target cells in the lung parenchyma in both early and later stages of infection, but SIV-infected macrophages were rare except in animals with lung interstitial inflammation defined by the increased infiltration of the pulmonary interstitium with a mixture of lymphocytes and plasma cells. This is also the case in HIV-1 infections where infected CD4+ T cells are rare outside of this inflammatory setting. The small number of infected macrophages in lung parenchyma, except in this setting, stands in stark contrast to the abundant SIV RNA+ cells in lymph nodes and gut where over 90% infected cells are CD4+ T cells as reported previously[13]. Moreover, SIV replication in CD4+ T cells peaks around 2 weeks post infection in secondary lymphatic tissues and gut mucosae [7], whereas virus replication in lung macrophages was very limited in this time frame (Table 1, Fig 1A1–1A2), consistent with a previous report[14]. We did find high levels of virus replication in lungs in the setting of lung interstitial inflammation, when virus replication in the lymphatic tissues and gut had already decreased. The increased infection of lung macrophages in this context cannot simply be attributed to target cell availability, as the macrophage population sizes were not different in early and later stages of infection and did not differ from uninfected controls. Rather, the milieu of lung interstitial inflammation may provide more permissive macrophage targets for viral replication in this context. However, interstitial inflammation is not frequent in SIV infections, which clearly sets these primate lentivirus infections apart from ungulate lentivirus infections such as maedi where interstitial pneumonitis is a defining pathological feature of these infections [15, 16]. Recently, lung macrophages have been classified into different subsets [17], and future studies are needed to test whether particular macrophage subset can preferentially support a high-level SIV replication in the setting of interstitial inflammation.

While severe CD4+ T cell depletion occurs in gut in the early HIV-1 infection [7, 18, 19], moderate CD4+ T cell depletion has been documented in bronchoalveolar lavage (BAL) [1–4], but the interstitial CD4+ cells in lung have not been quantified. Here, we show significant interstitial CD4+ T cell depletion in the lungs during very early infection that is sustained through 12 weeks. In contrast, macrophage populations do not decline despite the predominance of infection of macrophages in the lungs. There is thus no direct link between productive infection and population dynamics in the lungs. This disjunction between direct productive infection and depletion highlights the need for future studies to elucidate the mechanisms that account for CD4+ T cell depletion in the lungs and maintenance of macrophage populations even in the setting of direct infection. There are certainly precedents for redistribution of CD4 T cells as the mechanism underlying the apparent depletion in peripheral blood. Based on the recent demonstration that most CD8+ T cells isolated from mouse lung are actually confined to the pulmonary vasculature [20], we can envision altered dynamics of the flux between this pool and BAL as a mechanism accounting for apparent rapid CD4+ T cell depletion. Furthermore, the large numbers of SIV RNA+ macrophages in lungs in the setting of interstitial inflammation indicates that lung macrophages can be an important source for SIV persistent infection.
